# Thermo-Optical Sensitivity of Whispering Gallery Modes in As_2_S_3_ Chalcogenide Glass Microresonators

**DOI:** 10.3390/s22124636

**Published:** 2022-06-20

**Authors:** Alexey V. Andrianov, Maria P. Marisova, Elena A. Anashkina

**Affiliations:** 1 Institute of Applied Physics of the Russian Academy of Sciences, 46 Ulyanov Street, 603950 Nizhny Novgorod, Russia; marisova.mariya@rambler.ru (M.P.M.); elena.anashkina@ipfran.ru (E.A.A.); 2 Advanced School of General and Applied Physics, Lobachevsky State University of Nizhny Novgorod, 23 Gagarin Ave., 603022 Nizhny Novgorod, Russia

**Keywords:** microresonator with whispering gallery modes (WGMs), As_2_S_3_ chalcogenide glass microsphere, thermo-optical effect, thermo-optical sensitivity

## Abstract

Glass microresonators with whispering gallery modes (WGMs) have a lot of diversified applications, including applications for sensing based on thermo-optical effects. Chalcogenide glass microresonators have a noticeably higher temperature sensitivity compared to silica ones, but only a few works have been devoted to the study of their thermo-optical properties. We present experimental and theoretical studies of thermo-optical effects in microspheres made of an As_2_S_3_ chalcogenide glass fiber. We investigated the steady-state and transient temperature distributions caused by heating due to the partial thermalization of the pump power and found the corresponding wavelength shifts of the WGMs. The experimental measurements of the thermal response time, thermo-optical shifts of the WGMs, and heat power sensitivity in microspheres with diameters of 80–380 µm are in a good agreement with the theoretically predicted dependences. The calculated temperature sensitivity of 42 pm/K does not depend on diameter for microspheres made of commercially available chalcogenide fiber, which may play an important role in the development of temperature sensors.

## 1. Introduction

Dielectric microresonators with whispering gallery modes (WGMs) have plenty of diversified applications [1], among which sensing applications play a significant role [2,3,4,5,6,7,8,9,10,11]. Microresonators with certain advantages and disadvantages are produced from different materials using special technologies [12]. Among dielectric microresonators, glass microresonators are widely used, and silica ones are the most common since they can be manufactured in a reproducible manner from standard telecommunication fibers [13,14,15,16,17,18]. However, the use of other non-silica glasses with very different properties is also actively attracting the attention of different research groups since this allows expanding the boundaries of the possibilities and applications for microresonators and improving the characteristics of the devices based on them.

Chalcogenide glasses have been actively investigated for implementing nonlinear optical effects, since such glasses have huge cubic nonlinearities, ultrawide transparency ranges, and physical and chemical properties suitable for producing optical microresonators. For example, Raman lasing [19,20], optical parametric oscillations [21], the generation of Raman–Kerr optical frequency combs [22], and Brillouin generation [23] have been attained in chalcogenide microresonators.

Effects based on thermo-optical nonlinearity [24] have also been observed in chalcogenide microresonators. For instance, a laser wavelength in an Nd-doped chalcogenide microsphere was tuned when the pump power was changed; this fact was explained by the influence of nonlinear and/or thermal effects [25]. In [22], the tunability of a Raman wavelength was obtained with a change in temperature due to shifts of the WGM resonances. The start of Raman lasing was attained in [26] with the use of an auxiliary laser diode for microsphere heating to control the WGM shifts. The measurement of temperature sensitivity based on the same WGM wavelength shift effect with increasing ambient temperature in a chalcogenide microsphere was demonstrated in [11]. However, there have been almost no systematic studies of thermo-optical effects, including the investigation of temperature distributions in a microresonator during pump thermalization, thermal response times and the influence of these factors on the resonant wavelengths of WGMs, or on the influence of geometrical parameters on chalcogenide microresonators. However, such studies are very important for sensing applications, primarily for measuring ambient temperature as chalcogenide microresonators have higher sensitivity compared to, for example, silica ones [11]. Such research is useful in the search for an optimal design, choosing optimal parameters for sensor elements, and studying their limiting capabilities. Moreover, thermo-optical control using external heating or heating with an auxiliary laser diode can be used to quickly switch on/off nonlinear optical or laser generation and tuning the generated or transformed wavelengths.

Here we present detailed experimental and theoretical studies of thermo-optical effects in As_2_S_3_ microspheres. We investigated the temperature distributions caused by heating due to partial thermalization of the pump power and the corresponding wavelength shifts of WGMs. The temperature sensitivity and heat power sensitivity were studied.

## 2. Materials and Methods

### 2.1. Experimental Materials and Methods

**Figure 1:**
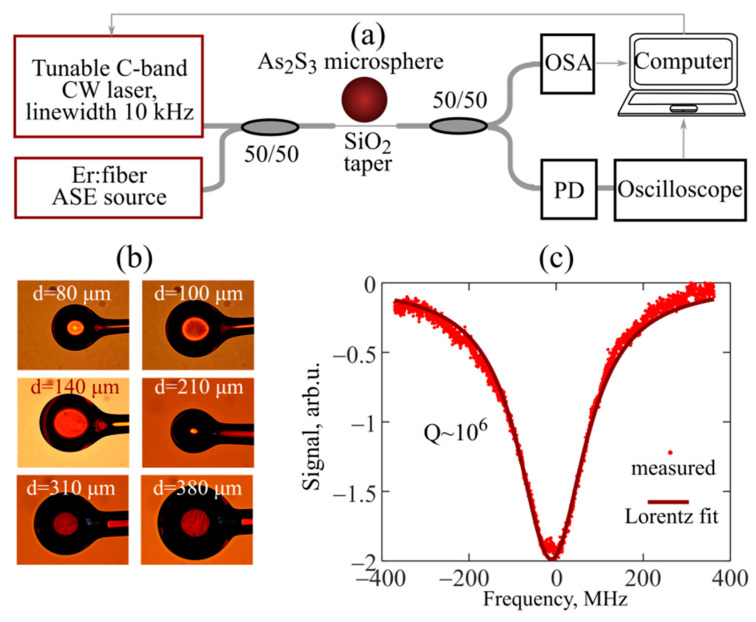
(**a**) Simplified scheme of the experimental setup; CW–continuous wave, ASE–amplified spontaneous emission, OSA–optical spectrum analyzer, PD–photodetector. (**b**) Images of experimental As_2_S_3_ chalcogenide glass microspheres obtained with optical microscope (with different magnification). (**c**) Measured resonance dip and its Lorentz fit demonstrating Q-factor.

The simplified experimental scheme is shown in Figure 1a. We performed studies with microspheres of different diameters, *d,* in the 80–380 µm range, made of a commercially available fiber based on As_2_S_3_ chalcogenide glass. Images of the microspheres used in all our experiments are presented in Figure 1b. The manufacturing method was based on melting the end of the fiber using a resistive microheater with the formation of a sphere under the action of surface tension forces. The details are given in our previous article [20]. CW pump laser radiation was coupled into the microsphere through a fiber taper made of a commercially available silica telecommunication fiber by heating and stretching [16,27]. We used a commercially available tunable telecommunication C-band laser (Pure Photonics, PLCC550-180-60) with a linewidth of 10 kHz and adjustable power up to 18 dBm (63 mW) supporting the frequency sweeping mode. The loaded quality factors (Q-factors) of microspheres were measured, and thermo-optical dynamic processes occurring during thermalization of the radiation power were investigated with the use of this laser. The central wavelength of the pump laser in all our experiments was set at about 1.55 μm.

The Q-factors of the microspheres were measured by the dynamic resonance scanning method, when the pump laser frequency was swept linearly at a rate of 10 GHz/s, and the resonant dip was recorded using an oscilloscope (Tektronix MSO-64 6-BW-2500, Beaverton, OR USA). The typical Q-factors for all samples, regardless of their diameter, were Q ~ 10^6^. An example of the measured data and the corresponding Lorentz fit are shown in Figure 1c. The pump power was attenuated to hundreds of nW to avoid nonlinear effects during measurements of the resonant dip.

In the study of thermo-optical effects, the pump laser power was set to be sufficiently high (~20–40 mW). Additionally, low-power broadband radiation from an erbium-doped fiber source of amplified spontaneous emission (ASE) was coupled with the microsphere to monitor WGM wavelengths. The radiation transformed in the microsphere was extracted through the same taper and then divided into two diagnostic channels. In one channel, the spectra were measured using an optical spectrum analyzer (OSA, Yokogawa AQ6370D, Tokyo, Japan), which allowed us to determine the WGM wavelength shifts. In the other channel, the signal was received by a photodetector (PD, Thorlabs PDA015C) and an oscilloscope, which made it possible to measure the transmitted power. When detuning of the pump laser frequency was changed, the absorbed power changed too, which could be estimated from the variations in the transmitted power. In these experiments, we did not observe nonlinear optical conversion of the pump frequency due to the Kerr and Raman nonlinearities, so we assumed that the absorbed power corresponded to the thermalized power. The pump laser was controlled by a computer. We processed and analyzed experimental data using specially developed algorithms and computer codes written on their basis.

### 2.2. Theoretical Model and Methods

To theoretically study thermo-optical processes and explain experimental results and dependences, we developed and applied a theoretical model based on finding the temperature distributions in As_2_S_3_ glass microspheres during pump power thermalization, followed by calculating the WGM eigenfrequencies and finding the wavelength shifts of WGMs Δ*λ* when changing the system parameters. In our earlier work, we used a similar model to study thermo-optical effects in tellurite glass microspheres [28].

**Figure 2:**
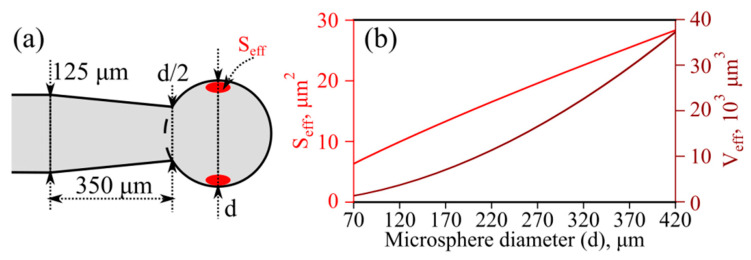
(**a**) Scheme of microresonator geometry used in simulations. (**b**) Effective mode area (red curve, left axis) and effective mode volume (brown curve, right axis) calculated at a wavelength of ~1.55 μm.

The microresonator geometry used in the numerical simulations is shown in Figure 2a. In our model, we assumed that pump thermalization occurs uniformly in a region near the sphere equator and the effective area *S*_eff_ and volume *V*_eff_ of this region are equal to the effective area and volume of the fundamental TE mode of the resonator, respectively, at a central wavelength of about 1.55 μm (Figure 2b). To calculate *S*_eff_ and *V*_eff_, we found the fields of the fundamental modes using expressions from [29].

To determine the steady-state and dynamic temperature distributions caused by partial thermalization of the pump power, we solved (by the finite element method) the heat equation numerically [30,31]:

(1) $$\rho c_{p}\frac{\partial \left(\Delta T\right)}{\partial t}+div\;q=Q,$$

(2) q=−k∇(ΔT),

where Δ*T* is the temperature increase relative to the ambient temperature, *ρ* is the As_2_S_3_ glass density, *c_p_* is the As_2_S_3_ glass heat capacity at constant pressure, *k* is the As_2_S_3_ glass thermal conductivity, ***q*** is heat flow, and *Q* is the heat source uniformly distributed over the volume *V*_eff_ (Figure 2).

The natural convection in air was set as a boundary condition on all surfaces, except for the end of the fiber opposite to the microsphere, where the temperature was fixed (although the boundary condition on this surface had practically no effect on the temperature distributions inside the microsphere). The boundary condition of natural convection was [30]:

(3) $$-\left(n_{\mathrm{norm}};q\right)=h\cdot \Delta T,$$

where ***n***_norm_ is a normalized outward-pointing vector, *h* ≈ 2 *k*_air_/*d* for a sphere, and *k*_air_ is the air thermal conductivity (a more precise expression for *h* used in our numerical simulations is given in Appendix A) [30]. Constants describing the material properties of As_2_S_3_ glass microspheres and other parameters are listed in Table 1.

To find the eigenfrequencies of the fundamental TE modes, we used the characteristic equation obtained from Maxwell’s equations based on the well-known approach [29]:

(4) $$\frac{{\left[{\left(nk_{0}d/2\right)}^{1/2}J_{l+1/2}\left(nk_{0}d/2\right)\right]}^{\prime }}{{\left(nk_{0}d/2\right)}^{1/2}J_{l+1/2}\left(nk_{0}d/2\right)}=\frac{1}{n}\frac{{\left[{\left(k_{0}d/2\right)}^{1/2}H_{l+1/2}^{\left(1\right)}\left(k_{0}d/2\right)\right]}^{\prime }}{{\left(k_{0}d/2\right)}^{1/2}H_{l+1/2}^{\left(1\right)}\left(k_{0}d/2\right)},$$

where *k*_0_ = 2π/*λ*, *c* is the speed of light in vacuum, *λ* is the wavelength, *d* is the microsphere diameter, *n* is the wavelength-dependent As_2_S_3_ glass refractive index (defined in Table 1), *l* is the polar WGM index, *J*_x_ is the Βessel function of order *x, H*_x_^(1)^ is the Hankel function of the 1st kind of order *x*, and the prime means the derivative with respect to the argument (*nk*_0_*d*/2 or *k*_0_*d*/2). Equation (4) has multiple roots corresponding to different radial WGM indices (*q* = 1 for the fundamental WGM). We numerically solved Equation (4) with allowance for the As_2_S_3_ glass dispersion and the dependence of *n* and *d* on the temperature increase

(5) $$n=n_{0}+\frac{dn}{dT}\Delta T_{\mathrm{mode}},$$

(6) $$\Delta d=d\varepsilon \Delta T_{\mathrm{av}},$$

where *n*_0_ is a linear refractive index; Δ*n* = (*dn*/*dT*)Δ*T*_mode_ and Δ*d* are changes in the refractive indexes of the As_2_S_3_ glass and microsphere diameters, respectively, under heating; *dn/dT* and *ε* are the thermo-optical and thermal expansion coefficients, respectively, of the As_2_S_3_ glass; and Δ*T*_mode_ and Δ*T*_av_ are the temperature increases averaged over the regions corresponding to the effective WGM size and the overall the sphere, respectively. Therefore, in our model we assumed that the temperature increase averaged over the sphere affects the thermal expansion, but Δ*n* is important only in the region where the fundamental WGMs are located.

## 3. Results

### 3.1. Theoretical Study of Temperature Distributions

**Figure 3:**
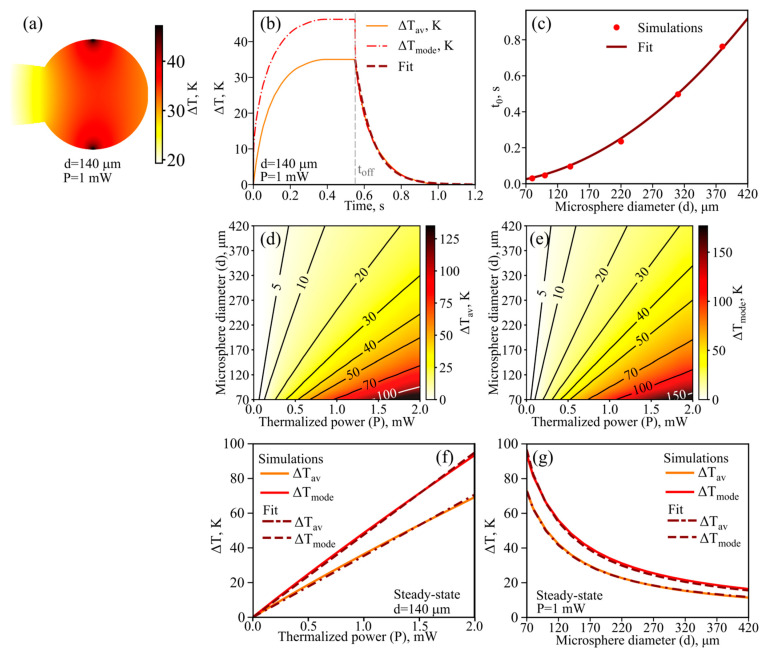
Numerical results. (**a**) Distribution of steady-state temperature increase in microresonator with diameter *d* = 140 µm for thermalized power *P* = 1 mW. (**b**) Temporal dynamics of temperature increases averaged over fundamental mode (Δ*T*_mode_) and over microsphere (Δ*T*_av_) when pump is switched on with a thermalized power of 1 mW and switched off at the moment *t*_off_, marked by the vertical gray dashed line. ‘Fit’ is approximation of Δ*T*_av_ by exponential decay with characteristic time *t*_0_ when pump is switched off. (**c**) Dependence of *t*_0_ on microsphere diameter obtained by direct simulations and fitted by *t*_0_ = *C*_1_⸱*d*^2^, where *C*_1_ = 5.2 × 10^−6^ s⸱µm^−2^. Steady-state temperature increase averaged over the microsphere (**d**) and over the fundamental mode (**e**) as functions of thermalized power and microsphere diameter. (**f**) Average temperature increases for *d* = 140 µm and their linear fits (Δ*T*~*P*). (**g**) Average temperature increases for *P* = 1 mW and their fits (Δ*T*~1/*d*).

First of all, we studied the temperature distributions arising in microspheres of different diameters upon partial thermalization of the pump power in the region corresponding to the effective size of the fundamental WGM at a wavelength of about 1.55 μm. As an example, the steady-state temperature distribution in a microsphere with a diameter of 140 μm is shown in Figure 3a. The maximum temperature is reached in the region where the heating source is located and decreases when moving away from the source. When the pump is switched on at the time *t* = 0, the microsphere begins to heat up; the curves for the average temperature increases Δ*T*_mode_ and Δ*T*_av_ are shown in Figure 3b. After a few hundred ms, the average temperatures reach steady-state values. After the pump is switched off at the moment of time *t*_off_, marked by the vertical dotted gray line, the microsphere begins to cool down, and the average temperatures relax to the ambient temperature (Figure 3b). The time dependence of the temperature decrease averaged over the sphere is well approximated by an exponential function with a characteristic time *t*_0_ (‘fit’ in Figure 3b):

(7) $$\Delta T_{\mathrm{av}}={\Delta T_{\mathrm{av}}|}_{t=t_{\mathrm{off}}}\cdot \exp\left(-\left(t-t_{\mathrm{off}}\right)/t_{0}\right).$$

We numerically simulated the dynamic cooling of microspheres of different diameters. The relaxation time *t*_0_ as a function of *d* is plotted in Figure 3c. Note that *t*_0_ is well approximated by the function *t*_0_ = *C*_1_⸱*d*^2^, where *C*_1_ = 5.2 × 10^−6^ s⸱µm^−2^ (‘fit’ in Figure 3c). Moreover, we found an analytical solution to the problem of cooling an ideal sphere with reasonable approximations, which gives an exponential decay for the average temperature and the dependence *t*_0_~*d*^2^. The found analytical solution allowed us to independently estimate the proportionality coefficient as 5.0 × 10^−6^ s⸱µm^−2^, which agrees very well with the numerically found coefficient *C*_1_. The analytical solution is given in Appendix A.

As for the temperature averaged over the WGM region, its dynamics are more complex, and within the framework of our model, are not described by a single decaying exponential dependence on time. Namely, when the pump is switched off, Δ*T*_mode_ first rapidly decreases to a value of about Δ*T*_av_ (*t* = *t*_off_) in time << *t*_0_, and then both average values Δ*T*_av_ and Δ*T*_mode_ decrease exponentially with a characteristic time *t*_0_ (Figure 3b).

Next, we calculated steady-state temperature distributions. The average temperatures increases Δ*T*_av_ and Δ*T*_mode_ as functions of a thermalized power, *P,* and a microsphere diameter, *d,* are presented in Figure 3d,e, respectively. Note that, for fixed *d,* these dependencies are almost linear (Δ*T*~*P*). The corresponding examples for *d* = 140 µm are plotted in Figure 3f. For fixed *P*, an average temperature increase is inversely proportional to the microsphere diameter. The corresponding examples for *P* = 1 mW are plotted in Figure 3g.

### 3.2. Theoretical Study of Steady-State Wavelength Shifts of the WGMs

**Figure 4:**
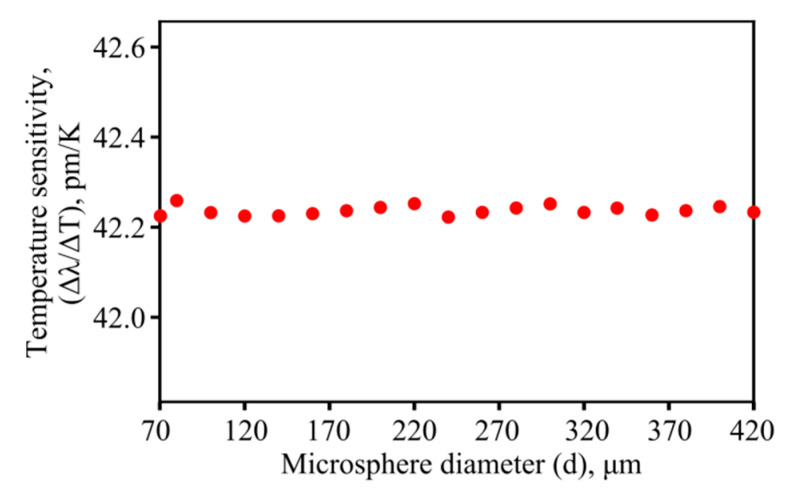
Numerically simulated steady-state sensitivity Δ*λ/*Δ*T* as a function of *d*.

Subsequently, we numerically simulated the wavelength shifts of the WGMs for a uniform increase in the temperature of the microsphere, which occurs with an increase in the ambient temperature. In this case, we assumed that the WGMs can be detected with the help of radiation of a very low power which does not affect the temperature distribution. The sensitivity Δ*λ*/Δ*T*, as a function of microsphere diameter, is shown in Figure 4. It is seen that the sensitivity is practically independent of the diameter, which is important for sensing. This peculiarity can be explained by using the resonance condition *l·λ = πdn_eff_*, where *n_eff_* is the WGM effective refractive index, which is primarily a function of *λ* but also weakly depends on the structure. Using Equations (5) and (6), an approximate relation between Δ*λ* and Δ*T* can be obtained:

(8) $$\Delta \lambda \approx \frac{\pi d}{l}\left(\varepsilon n_{eff}\Delta T+\frac{dn}{dT}\Delta T\right)\approx \lambda \left(\varepsilon +\frac{1}{n_{eff}}\frac{dn}{dT}\right)\Delta T.\;$$

Therefore, Δ*λ*/Δ*T* is almost a constant value for modes near a given wavelength. Namely, when using As_2_S_3_ chalcogenide microspheres having diameters from several tens to several hundreds of microns, the predicted sensitivity is ~42 pm/K. However, small perturbations of temperature sensitivity are observed for microresonators of different sizes (Figure 4). The explanation is that the eigenfrequencies and corresponding *n_eff_* found by solving Equation (4) are slightly varied for the fundamental WGMs (nearest to 1.55 µm) in microspheres with different diameters.

**Figure 5:**
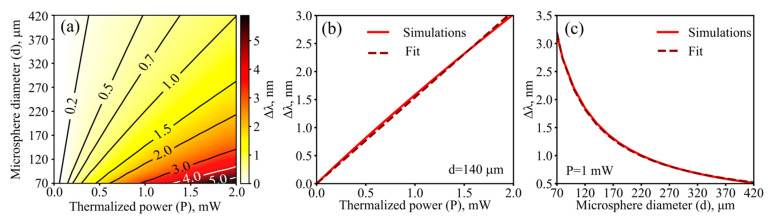
Numerical results. (**a**) Steady-state thermo-optical shift of WGMs, Δλ, as a function of thermalized power and microsphere diameter. (**b**) Δ*λ* as a function of *P* for *d* = 140 µm and its linear fit. (**c**) Δ*λ* as a function of *d* for *P* = 1 mW and its fit ~1/*d*.

Next, we studied the wavelength shifts of the WGMs occurring as a result of temperature increase due to the thermalization of the pump power. The numerically simulated Δ*λ* as a function of *P* and *d* is shown in Figure 5a. For a microsphere of a fixed size, Δ*λ* is an almost linear function of *P*. The example for *d* = 140 µm is plotted in Figure 5b. Moreover, at a fixed power, Δ*λ* is inversely proportional to *d* (Figure 5c).

### 3.3. Experimental and Theoretical Study of Temporal Dynamics of the Wavelength Shifts of the WGMs

We investigated the temporal dynamics of the WGM wavelength shifts after the pump was switched off. In this experiment, a steady-state temperature distribution was established under the action of a CW pump and the WGM wavelengths had certain values. Then, the pump was switched off, after which the microsphere cooled down and the WGM wavelengths shifted to the short-wavelength range.

**Figure 6:**
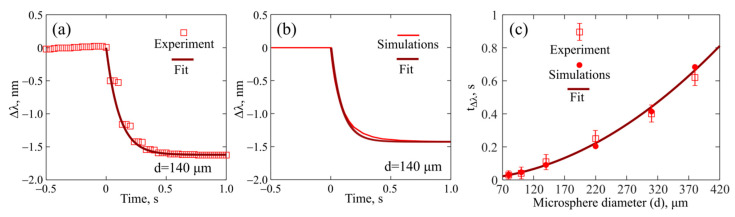
Experimental (**a**) and numerically simulated (**b**) dependences of thermo-optical shift in WGMs Δλ at time *t*. Thermalized power is 0.9 mW for *t* < 0 and the pump is switched off at *t* = 0. ‘Fit’ is approximation of Δλ by exponential decay with characteristic time *t*_Δλ_ for *t* > 0; (**c**) *t*_Δλ_ dependence on microsphere diameter obtained by experimental measurements, by direct simulations and fitted by *t*_Δλ_ = *C*_2_⸱*d*^2^, where *C*_2_ = 4.6 × 10^−6^ s⸱µm^−2^.

We measured the temporal dynamics of Δ*λ* for all samples of the produced microresonators. An example of the measurements for a microresonator with a diameter of 140 μm is shown in Figure 6a. The pump was switched off at *t* = 0. The decreasing function Δ*λ*(*t*) can be approximated by the exponential dependence on time

(9) $$\Delta \lambda ={\Delta \lambda |}_{t=0}\cdot \exp\left(-t/t_{\Delta \lambda }\right).$$

We performed direct simulations in the framework of the model described in Section 2.2 and obtained the numerical dependence of Δ*λ* for *t* after the pump was switched off. An example of Δ*λ*(*t*) for *d* = 140 µm is plotted in Figure 6b. Experimental and numerical results agree very well (compare Figure 6a,b). However, in our theoretical model, Δ*λ*(*t*) is not an ideal exponential function because Δ*λ* depends on both Δ*T*_av_ and Δ*T*_mode_. The explanation based on the results obtained in Section 3.1 is that Δ*T*_av_ is well-fitted by an exponential dependence, but Δ*T*_mode_ is a more complex function. Consequently, the simulated values of *t*_Δλ_ are slightly lower than *t*_0_ (the difference is about 10%). The experimentally measured and numerically simulated *t*_Δλ_ values for different microsphere diameters are shown in Figure 6c. The corresponding approximation *t*_Δλ_ = *C*_2_⸱*d*^2^, where *C*_2_ = 4.6 × 10^−6^ s⸱µm^−2^ is also plotted.

### 3.4. Experimental and Theoretical Study of Δλ/P

**Figure 7:**
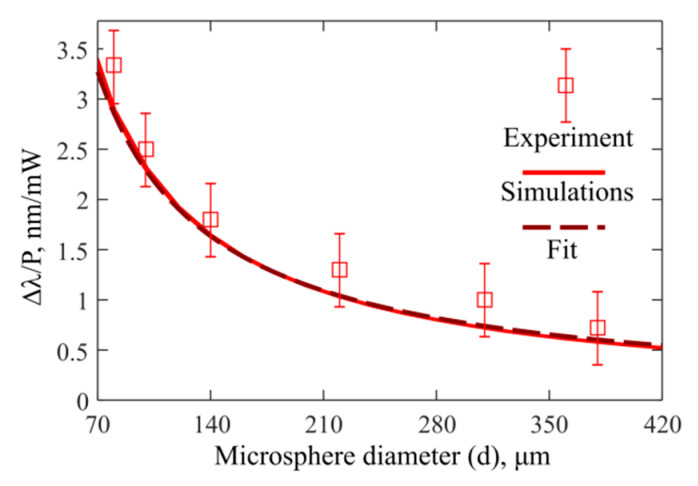
Heat power sensitivity Δ*λ*/*P* versus microsphere diameter obtained by experimental measurements, by direct simulations, and fitted by Δ*λ*/*P* = *C*_3_/*d*, where *C*_3_ = 230 nm⸱µm/mW.

Finally, we measured the heat power sensitivity Δ*λ*/*P* for all produced microspheres (Figure 7). The simulations demonstrate that Δ*λ*/*P* is proportional to 1/*d*. Note that the presented experimental data and numerical dependences agree very well. The main sources of uncertainties in our measurements are the OSA resolution of 0.02 nm, its limited scanning speed, and some drifts in the photodetector signal used for power measurements. For the microspheres with a high density of WGMs, wavelength tracking errors may introduce additional uncertainty into the WGM thermal shift measurements larger than the OSA resolution.

## 4. Discussion

We produced chalcogenide glass microspheres with diameters of 80–380 µm and typical Q-factors of 10^6^ from a commercially available As_2_S_3_ fiber and investigated their thermo-optical properties experimentally and theoretically. The theoretical studies were based on numerical simulations of the heat equation using the finite-element method for experimental-like geometry. Average temperature increases were calculated and taken into account aimed at finding the eigenfrequencies of the WGMs and thermo-optical shifts caused by thermal expansion of the microsphere and the temperature dependence of the refractive index. We investigated the steady-state and transient temperature distributions caused by heating due to the partial thermalization of the pump power and found the corresponding wavelength shifts of the WGMs. We showed theoretically that average steady-state temperature increases are proportional to the thermalized pump power and inversely proportional to the microsphere diameter. We determined experimentally, numerically, and even analytically that the thermal response time after switching off the pump is proportional to the microsphere diameter squared, and the coefficient is about 5 × 10^−6^ s⸱µm^−2^.

We demonstrated through numerical simulations that for As_2_S_3_ chalcogenide microspheres the temperature sensitivity Δ*λ*/Δ*T* = 42 pm/K does not depend on diameters ranging from several tens to several hundreds of microns, and a simple explanation in terms of the effective refractive index of the mode was provided. Note that this sensitivity is higher than that for microspheres made from many other glasses. For example, for a gallium–germanium–antimony–sulfide chalcogenide glass microsphere with a diameter of about 100 µm, the measured sensitivity was 28 pm/K [11]. For silica microspheres, the experimental sensitivity at room temperature was about 10 pm/K and was almost independent of the diameter [10]. In addition, although we did not directly measure Δ*λ/*Δ*T* for an external increase in ambient temperature, we measured heat power sensitivity Δ*λ*/*P* and obtained good agreement with the theoretically predicted dependence Δ*λ*/*P~1/d*. Therefore, this fact confirms that our numerical model is well calibrated and gives reliable results.

The microresonators studied here can serve as microheaters and sensors simultaneously, while the measurement of the WGM shifts provides quite accurate temperature readings. Chalcogenide glasses ensure extended transparency in the mid-IR range, where the spectral fingerprints of many important chemical substances are located. The chalcogenide glass–based microresonators may be used as multipurpose devices combining heating/temperature-measuring functionalities and spectral sensing of their environment. Currently, fibers based on chalcogenide As_2_S_3_ glass are commercially available, so microsphere manufacturing based on them can be a routine, fast, and inexpensive process. The independence of temperature sensitivity and microsphere diameter means that it is not necessary to control the diameter of the samples for such sensors. Thus, the demonstrated results are quite universal and may be practically significant.

## Figures and Tables

**Table 1:** Parameters used in simulations.

Parameter			Value	
As_2_S_3_ glass thermal conductivity (*k*)			0.17 W/(m·K) [32]	
As_2_S_3_ glass density (*ρ*)			3.20 gm/cm³ [32]	
As_2_S_3_ glass heat capacity at constant pressure (*c_p_*)			460 J/(kg·K) [32]	
As_2_S_3_ glass thermo-optical coefficient (*dn/dT*)			9·10^−6^ K^−1^ [33]	
As_2_S_3_ glass thermal expansion coefficient (*ε*)			25·10^−6^ K^−1^ [34]	
Air thermal conductivity (*k*_air_)			0.025 W/(m·K)	
Refractive index (*n*_0_). $n_{0\;}^{2}=1+\sum_{i=1}^{i=5}\frac{A_{i}\lambda^{2}}{\lambda^{2}-B_{i}}$ [33] (*A*_i_ and *B*_i_ are Sellmeier constants)				
*A_1_*	*A_2_*	*A_3_*	*A_4_*	*A_5_*
1.8983678	1.9222979	0.8765134	0.1188704	0.9569903
*B_1_*, μm^2^	*B_2_*, μm^2^	*B_3_*, μm^2^	*B_4_*, μm^2^	*B_5_*, μm^2^
0.0225	0.0625	0.1225	0.2025	750

## Data Availability

Data underlying the results presented in this article may be obtained from the authors upon reasonable request.

## Appendix A

Here, we provide the analytical solution to the problem of the cooling of an ideal microsphere (without supporting fiber stem) and directly show the temperature relaxation time dependence on diameter *t_0_* ~ *d^2^* and estimate the corresponding constant. The convection coefficient *h* (as in Equation (3)) for external natural convection on a sphere used in our simulations is given by an empirical relation (Equation (9.35) in [30]):

(A1) $$h=\frac{k_{air}}{d}\left(2+\frac{0.589Ra_{d}^{\frac{1}{4}}}{{\left(1+{\left(\frac{0.469}{Pr}\right)}^{\frac{9}{16}}\right)}^{\frac{4}{9}}}\right),\;$$

(A2) $$Ra_{d}=\frac{g\varepsilon_{p,\;air}\rho_{air}^{2}c_{p,\;air}\left|\Delta T\right|d^{3}}{k_{air}\mu_{air}}.$$

Here *Ra_d_* is the Rayleigh number for a sphere, *Pr = μ_air_·c_p,air_/k_air_* = 0.72 is the Prandtl number for air, *g* = 9.81 m/s^2^ is the acceleration due to gravity, *ε_p,air_* = 3.43·10^−3^ K^−1^ is the thermal expansion coefficient for air, *ρ_air_* = 1.2 kg/m^3^ is the air density, *μ_air_* = 1.83·10^−5^ kg/(m·s) is the air dynamic viscosity, and *c_p,air_* = 1007 J/(kg·K) is the air heat capacity at constant pressure. For our analytical solution, we neglected the temperature dependence of the air parameters (although it was taken into account in our simulations). The second term in the parentheses in Equation (A1) can be estimated for the considered microsphere diameters, *d,* and the steady-state temperature increases, Δ*T_av_* (obtained in Section 3.1). The maximum estimate for the term yields approximately 0.25; this is significantly smaller than 2. Therefore, we neglected it in our analytical model and took *h ≈ 2k_air_/d*.

Furthermore, we assumed the temperature distribution to have spherical symmetry during cooling. By rewriting the heat Equation (1) and the boundary condition (3) in spherical coordinates (*r*, *θ*, *φ*) and neglecting any dependencies on (θ, φ), we obtained:

(A3) $$\frac{\rho c_{p}}{k}\frac{\partial \Delta T}{\partial t}=\nabla^{2}\left(\Delta T\right)=\frac{1}{r^{2}}\frac{\partial }{\partial r}\left(r^{2}\frac{\partial \Delta T}{\partial r}\right),$$

(A4) $${\frac{\partial \Delta T}{\partial r}|}_{r=d/2}=-\frac{2k_{air}}{kd}{\Delta T|}_{r=d/2}.$$

The coordinate system center (*r* = 0) corresponds to the sphere center. Then we followed the separation of variables method (also known as the Fourier method) for solving partial differential equations. We made the following substitution:

(A5) ΔT(r, t)=u(r)f(t)

Inserting Equation (A5) into Equations (A3) and (A4) yields:

(A6) $$\frac{\rho c_{p}}{k}u\left(r\right)f^{\prime }\left(t\right)=f\left(t\right)\frac{1}{r^{2}}\frac{d}{dr}\left(r^{2}u^{\prime }\left(r\right)\right),$$

(A7) $${\frac{du}{dr}|}_{r=d/2}=-\frac{2k_{air}}{kd}{u|}_{r=d/2},$$

where the prime denotes the derivative. Dividing both sides of Equation (A6) by *u*(*r*)*·f*(*t*) provides the following relation:

(A8) $$\frac{\rho c_{p}}{k}\frac{f^{\prime }\left(t\right)}{f\left(t\right)}=\frac{\frac{1}{r^{2}}\frac{d}{dr}\left(r^{2}u^{\prime }\left(r\right)\right)}{u\left(r\right)}=-\eta .$$

As the left-hand side of Equation (A8) depends only on *t*, and the middle part depends only on *r*, both these expressions are equal to the constant *–η*. We are interested in a cooling process, *f*′(*t*) < 0, therefore, *η* > 0. Equation (A8) is now reduced to two ordinary differential equations for *u*(*r*) and *f*(*t*). Their corresponding analytical solutions can be obtained directly (assuming *t* = 0 to be the beginning of the cooling process):

(A9) $$f\left(t\right)=f_{0}\exp\left(-t\frac{k\eta }{\rho c_{p}}\right),$$

(A10) $$u\left(r\right)=\frac{u_{01}}{r}\sin\sqrt{\eta }r+\frac{u_{02}}{r}\cos\sqrt{\eta }r,$$

where *f*_0_, *u*_01_, and *u*_02_ are arbitrary constants. However, *u*_20_ = 0 as, otherwise, *u(r→0)→∞*. From Equation (A9) it is obvious that the previously introduced temperature relaxation time in Equation (7) is *t*_0_ *= ρ·c_p_*/(*k·η*) and its dependence on microsphere diameter is determined by *η*. Using the obtained solution *u*(*r*) in the boundary condition (A7) yields:

(A11) $$\sqrt{\eta }\frac{d}{2}=\left(1-\frac{k_{air}}{k}\right)\tan\sqrt{\eta }\frac{d}{2},\;$$

From here it is obvious that *η = (2ξ/d)^2^*, where *ξ* is a root of *ξ* ≈ 0.8521·tan *ξ*. The smallest root *ξ* ≈ 0.656 was chosen, as it corresponds to the slowest cooling harmonic (without any radial variations in Δ*T*), which is most likely to occur in our system. The final expression for *t_0_* (in seconds) is:

(A12) $$t_{0}\left[s\right]=\frac{\rho c_{p}}{4\xi^{2}k}d^{2}\approx 5.0\cdot 10^{-6}{\left(d\left[\mu m\right]\right)}^{2}$$
